# Household Air Pollution from Biomass Fuel for Cooking and Adverse Fetal Growth Outcomes in Rural Sri Lanka

**DOI:** 10.3390/ijerph18041878

**Published:** 2021-02-15

**Authors:** Alicia Vakalopoulos, Shyamali C. Dharmage, Samath Dharmaratne, Pasan Jayasinghe, Olivia Lall, Isabella Ambrose, Rohan Weerasooriya, Dinh S. Bui, Duminda Yasaratne, Jane Heyworth, Gayan Bowatte

**Affiliations:** 1 Allergy and Lung Health Unit, Melbourne School of Population and Global Health, University of Melbourne, Carlton, VIC 3053, Australia; avakalopoulo@student.unimelb.edu.au (A.V.); olall@student.unimelb.edu.au (O.L.); iiambrose@student.unimelb.edu.au (I.A.); dinh.bui@unimelb.edu.au (D.S.B.); gayan.bowatte@unimelb.edu.au (G.B.); 2 Department of Community Medicine, Faculty of Medicine, University of Peradeniya, Peradeniya 20400, Sri Lanka; samath.dharmaratne@mbbs.md; 3 Department of Health Metrics Sciences, Institute for Health Metrics and Evaluation, School of Medicine, University of Washington, Seattle, WA 98195, USA; 4 Department of Health Services, Central Province, Kandy 20000, Sri Lanka; pasan978@gmail.com; 5 National Institute of Fundamental Studies, Hantana Road, Kandy 20000, Sri Lanka; rohan.we@nifs.ac.lk; 6 Department of Medicine, Faculty of Medicine, University of Peradeniya, Peradeniya 20400, Sri Lanka; yasaratne@yahoo.com; 7 School of Population and Public Health, Faculty of Health and Medical Sciences, University of Western Australia, Nedlands, WA 6009, Australia; jane.heyworth@uwa.edu.au; 8 Department of Basic Sciences, Faculty of Allied Health Sciences, University of Peradeniya, Peradeniya 20400, Sri Lanka

**Keywords:** household air pollution, indoor air pollution, biomass, pregnancy, Sri Lanka, LBW, SGA

## Abstract

The aim of this study was to investigate the impact of biomass fuel for cooking on adverse fetal growth outcomes in Sri Lanka. A cross-sectional study of mothers recruited at maternity clinics in rural communities in Sri Lanka’s Central Province was undertaken. Data pertaining to household air pollution and fetal growth parameters were collected using an interviewer-administered questionnaire. Logistic regression models, adjusted for potential confounders, were used to evaluate the impact of biomass fuel for cooking on low birth weight (LBW) and small for gestational age (SGA) parameters. Findings showed that exposure to biomass cooking fuels during pregnancy was associated with an increased risk of LBW adjusted odds ratio (aOR) 2.74 (95% CI 1.08–6.96) and SGA (aOR: 1.87, 95% CI 1.03–3.41) compared with the use of clean energy. The risk of LBW was highest for traditional biomass stoves compared to improved biomass stoves (aOR: 3.23, 95% 1.17–8.89) and biomass use in kitchens without a chimney compared to kitchens with a chimney (aOR: 4.63, 95% 1.54–13.93). Similar trends were observed for SGA.

## 1. Introduction

Household air pollution (HAP) accounts for 3% of global deaths each year and 6% in low- and middle-income countries (LMICs) [1]. HAP is attributed primarily to the burning of solid fuels such as biomass for cooking [1]. Over three billion people rely upon solid fuels, including biomass, as their primary source of domestic energy worldwide [1]. Biomass refers to organic material derived from plants and animals which may be burnt to produce energy [2]. Compared to clean energies such as liquid petroleum gas (LPG), biomass fuels release higher concentrations of carbon monoxide (CO) and fine particulate matter (particulate matter < 2.5 μm in diameter (PM_2.5_)) into household environments [3]. The majority of biomass users reside in LMICs, where cost reduces access to clean energy alternatives [4].

In Sri Lanka, biomass-fuelled stoves are the leading contributor to HAP [5,6]. Sixty-six percent of Sri Lankan households opt for biomass, principally firewood, as their main source of cooking fuel [7]. Wood burning commonly occurs indoors with thermally inefficient, traditional mud stoves and in households with poor ventilation [8]. While the consequences of HAP are manifold, there is a growing body of evidence linking exposure with adverse antenatal outcomes such as low birth weight (LBW) and fetuses small for gestational age (SGA) [9,10,11,12,13,14,15,16,17,18,19]. Only two studies have been conducted on the association between HAP and LBW, and none on SGA in Sri Lanka to date [9,20]. The aim of this study was to investigate the association between HAP and adverse fetal growth outcomes considering primary and secondary fuel use, stove-type, and ventilation in two rural Sri Lankan communities.

## 2. Materials and Methods

### 2.1. Study Design

A cross-sectional study was undertaken using interviewer-administrated questionnaires to collect primary data on exposure, outcome and confounder variables. Questionnaires were written in English by the primary study investigators, then translated into Sinhalese and Tamil. Questions pertaining to HAP exposure were adjusted from the BOLD Core questionnaire [21]. The final survey instrument enabled many facets of HAP to be addressed simultaneously, such as choice of cooking fuel, stove type, and kitchen ventilation. Antenatal health was measured by questions formulated from birth cards including birthweight and gestational age at birth. Birth cards are provided to all pregnant mothers for each of their children at Ministry of Health (MoH) clinics nationwide.

### 2.2. Study Population

The source population comprised women with a child under 5 who attended a vaccination clinic at either the Udunuwara or Gampola health clinics, which are located in rural Medical Officer of Health (MoH) areas in Sri Lanka’s Central Province. The total population of the two MoH areas is estimated to be 174,000 inhabitants [22]. MoH areas were selected based on them having rural and communities who were generally from a low socio-economic background. The urban community sampled was located in Gampola and housed those working or living in Sri Lanka’s tea and rubber plantations. This population comprised a comparatively low socio-economic class.

### 2.3. Recruitment and Sampling

Recruitment occurred during child vaccination programs and routine antenatal examinations in antenatal clinics in the selected MoH areas. Mothers who attended were approached to determine eligibility. Mothers were eligible if they had at least one child aged five or under. Prospective participants were provided with detailed information on the study, and requirements of participation including a copy of the Plain Language Statement. All information and documents were provided in the primary language of the study participant. Appointments for home-visits were then scheduled. A convenience sample of 395 households was achieved, with a response rate of 100%. Out of the 24 clinics in this area, the main three clinics, which the majority of the mothers attended for vaccination, were selected to obtain a representative sample.

### 2.4. Data Collection

Data collection took place over five weeks between August and September 2019. Questionnaires were administered by pre-intern medical students from the University of Peradeniya. Questions focused on household fuel use, stove type, ventilation, and sources of HAP other than cooking (smoking, mosquito coil, incense burning and vaporisers). Questions were repeated for each pregnancy for mothers who had more than one child in the past six years. The recall period of six years was acceptable given the stability of cooking practices in the region [2]. Data on socio-demographic characteristics, including household income and mother’s education, were collected. Informed consent was obtained from each mother. Ethical approval was granted by the Faculty of Allied Health Sciences at the University of Peradeniya (AHS/ERC/2019/021, 30 July 2019).

### 2.5. Exposure Assessment

Three different measures were used to assess exposure to HAP during pregnancy. The first method compared the primary sources of cooking fuel, “biomass” and “clean energy—mainly LPG”. The second method compared stove type: “traditional biomass stove” and “improved biomass stove” to “clean energy stove”. The third method categorised HAP according to primary and secondary fuel type and ventilation. Many households used more than one type of fuel for cooking. Primary fuel referred to the fuel type most frequently employed. Women utilising biomass as their primary fuel in kitchens without a chimney were categorised as “very high” exposure; those using biomass as their primary fuel in kitchens with a chimney were categorised as “high” exposure; and “moderate” exposure was specific to households using biomass as a secondary source to clean energy. Those using only clean energy formed the “low” exposure reference category. Clean energy refers to electricity, biogas, or most commonly LPG.

### 2.6. Outcome Assessment

Antenatal outcome data were obtained from birth cards. In Sri Lanka, birth cards are provided to all mothers at the time of pregnancy, and include information pertaining to key maternal and child health indices. The selected study outcomes were specific to two indicators of fetal growth: LBW and SGA. Birth cards provided information on the birth weight of the child in kilograms, recorded at the hospital by a nurse or midwife immediately after birth. Children were then categorised as being of LBW if they were born at a weight less than 2.50 kg. Children were categorised as being SGA if they were born at a weight below the 10th percentile for a particular gestational age. SGA was determined using the World Health Organization’s (WHO) internationally applicable fetal growth calculator [23]. Surveyors were able to review the birth cards of 100% of pregnancies.

### 2.7. Statistical Analysis

Analysis was undertaken using the STATA 15.1 (Stata Corporation, College Station, TX, USA) statistical program for both descriptive and inferential statistics. Descriptive statistics were used to summarise socio-demographic and other baseline characteristics. Logistic regression models were then used to measure the impact of HAP on LBW and SGA. Models were adjusted for confounders and clustered with respect to household to account for mothers with multiple children aged 5 or under. The most relevant confounders to the Sri Lankan context were identified using a directed acyclic graph (DAG) (Supplementary Figure S1) and are supported by the current literature [2,8,9,20,24]. These were household monthly income, education, area, the use of incense, the use of a vaporiser for mosquitos, and exposure to second-hand tobacco smoke. Consistent with published literature, ventilation was included in the models for exposure assessment methods 1 and 2 described previously, and was measured by the presence or absence of a chimney [25].

## 3. Results

Data were collected for 245 rural households in Udunuwara, 65 households in Gampola, and 74 households in the urban sector in Kandy district in Sri Lanka. Incomplete questionnaire responses meant limited or no data on childhood outcomes and resulted in the exclusion of five households from the analysis. A further six households were excluded due to multiple births: five cases of twins and one case of triplets. The final dataset included information on 445 live births in the past five years, 385 mothers from 384 households.

### 3.1. Descriptive Statistics

#### 3.1.1. Demographics

The rural areas of Udunuwara and Gampola were home to 81% of study participants, with 45% in households using biomass fuel. The remaining 19% of participants livedated in urban communities, where the prevalence of biomass use was 64%. The primary profession for three out of four mothers was housewife. For households earning over LKR 75,000 (USD 414) each month, 75% opted for non-polluting gas or electric stoves. This is compared to 39% for households with monthly earnings of under LKR 25,000 (USD 137) (Table 1).

#### 3.1.2. Preference for Stove and Fuel Type

The proportion of households primarily using biomass or clean energy (LPG or electricity) was 48% and 52%, respectively. All biomass-using households opted for firewood, while for 22% of these households, almost half of biomass users, supplemented firewood with the husks and the shells of coconuts. Among biomass-using households, 55% used improved mud stoves and 45% used traditional mud stoves. LPG was used in 51% of households. The majority of households (86%) employed a secondary source of cooking fuel. Similar trends in the distribution of clean energy compared to biomass was observed for secondary fuel use, although electricity demonstrated greater use, in 20% of all households (Table 2). The majority of households using clean energy (LPG or electricity) as their primary source of fuel opted for biomass as their secondary source. The majority of households using biomass as their primary source of fuel opted for clean energy (LPG or electricity) as their secondary source (Supplementary Table S3).

#### 3.1.3. Cooking Practices, Kitchen Characteristics and Maternal Health Indices

Sixty percent of mothers devoted 2–3 h to cooking each day during the pregnancy period, and a further 29% cooked for more than three hours daily. Four out of five mothers engaged in cooking during all trimesters of pregnancy. Thirty percent of households that used biomass as the primary source of fuel did not have a chimney (Supplementary Table S1).

The prevalence of LBW was 13%, of which 59% were exposed to biomass emissions in utero. The prevalence of SGA was 43%, and children who were born pre-term represented 7.5% of the sample. The average birth weight was 2.92 kg, and the average gestational period was 38.8 weeks (Supplementary Table S2).

### 3.2. The Association between HAP and Adverse Fetal Growth

#### 3.2.1. The Association between HAP and LBW

Mothers using biomass for cooking during pregnancy had an increased chance of delivering a child with LBW compared to those using clean energies (adjusted odds ratio (aOR) 2.74 (95% CI 1.08–6.96). Mothers using traditional biomass stoves had an increased risk of giving birth to a child with LBW compared to those using clean energy (aOR 3.23 (95% CI 1.17–8.89)). An increased risk was also seen between improved biomass stoves and LBW, but this was not statistically significant (aOR 2.11 95%CI (0.80–5.53)) (Table 3).

Very high HAP exposure (biomass as a primary fuel source in a no-chimney kitchen) resulted in an increased risk of LBW compared to the low exposure reference category (clean energy as only source of cooking fuel), with an aOR of 4.63 (95% CI 1.54–13.93) (Table 3). As exposure to HAP increased from low to very high, so did the risk of LBW (*p*-value for overall trend = 0.005) (Table 3).

#### 3.2.2. The Association between HAP and SGA

Mothers’ exposure to biomass as their primary source of cooking fuel was associated with an increased risk of giving birth to a child SGA compared to those using clean energy aOR of 1.87 (95% CI 1.03–3.41) (Table 4). Mothers’ exposure to traditional biomass stoves was also associated with an increased risk of giving birth to a child SGA compared to those using clean energy stoves aOR of 2.64 (95% 1.27–4.91) (Table 4).

Very high exposure (biomass as a primary fuel without chimney) and high exposure (biomass as a primary fuel with chimney) was not associated with SGA. However, as exposure increased from low to very high, so did the risk of SGA. The *p*-value for this overall trend was 0.0199 (Table 4).

## 4. Discussion

Results from this study add to the current body of evidence that biomass fuel compared to clean energy for cooking impairs fetal growth, increasing the risk of both LBW and SGA. It is one of the few Sri-Lankan-specific studies assessing the impact of biomass on LBW and the first of its kind on SGA. Compared to other LMICs, Sri Lanka’s low prevalence of competing antenatal risk factors, coupled with a high prevalence of biomass fuel makes the country an ideal study location [26]. In fact, the country achieves 100% provision of antenatal care to its citizens, and 99.5% of mothers receive skilled attendance during childbirth, standards comparable to the developed world [26,27]. Sri Lanka also has the lowest levels of ambient air pollution in South Asia [28].

Although only one other questionnaire-based investigation on the impact of wood-fuel on LBW in Sri Lanka exists to date, the results show a similar effect size to this study [20]. Comparable trends are also evident in various Indian studies [13,14] and in a meta-analysis of 19 studies, in which the use of solid fuel was associated with a 35% (95% CI: 23, 48) increased risk of LBW in LMICs [11]. In a study in Sri Lanka’s Western Province, a 10-fold increase in household PM_2.5_ was associated with a 54% (aOR: 1.54, 95% CI: 1.12, 2.12) increased risk of LBW [9].

Current literature further supports findings from this study, that poor kitchen ventilation and traditional biomass stoves increase exposure to HAP, heightening the risk of adverse antenatal outcomes. According to the WHO, the target level for indoor PM_2.5_ is 25 µg/m^3^ [29]. A study of 53 biomass-using rural households in Sri Lanka’s Western Province found 48 h averaged PM_2.5_ concentrations of 70 µg/m^3^ in the presence of a chimney and 371 µg/m^3^ without a chimney [24]. In another Sri Lanka study, personal exposure to PM_2.5_ was substantially lower for improved biomass stoves at a range of 83–114 µg/m^3^ compared to traditional biomass stoves at 139–215 µg/m^3^ [30].

### 4.1. Pathological Mechansisms

PM_2.5_ and CO act through various mechanisms to alter fetal health. CO binds to haemoglobin to reduce the oxygen-carrying capacity of the blood [31]. In pregnant women, this may lead to insufficient oxygen delivery to the fetus and fetal growth retardation, increasing the risk of both LBW and SGA [32]. CO is also a fetotoxin, meaning it can cross the placental barrier and act directly upon the fetus during a critical stage of development [11,32]. Physiological immaturity and elevated rates of cell proliferation makes the fetus highly sensitive to external factors [11,33].

PM_2.5_ penetrates the alveoli sacs to trigger oxidative stress and induce systemic inflammation [34]. A study of pregnant women in Nigeria found a positive association between indoor PM_2.5_ and elevated levels of the inflammatory marker TNF-a. This was reduced following a switch to clean energy [35]. A meta-analysis also found associations between prenatal exposure to PM_2.5_ and chromosomal abnormalities, notably DNA methylation of CpGs [34]. Chromosomal abnormalities have been linked to fetal growth restriction and LBW [36,37]. It is therefore possible that oxidative stress-induced epigenetic changes contribute to adverse antenatal aetiologies [38].

### 4.2. Strengths and Limitations

A key strength of this study was the incorporation of a wide range of HAP determinants into three different exposure models, including primary and secondary fuel use, stove type, and kitchen ventilation. In contrast, previous studies commonly limited exposure assessment to primary fuel type. Over 78% of Sri Lankan households use more than one source of cooking fuel; therefore, disregarding secondary fuel use is likely to result in residual confounding and underestimate the impact of HAP on LBW [9].

Although household questionnaires were useful in the consideration of many facets of HAP, the approach did not enable exact indoor concentrations to be measured. Using monitors to record household concentrations of PM_2.5_ and CO presents a more precise although expensive method to measure HAP. Studies that have employed this approach drew conclusions consistent with results from this study [9,14].

Despite secondary fuel being considered in analysis, no data were collected regarding the proportionate use of secondary fuel compared to primary fuel. Such distinctions would be useful to increase the accuracy of HAP measurements using a questionnaire-based approach. Exposure data relied upon maternal recall, and although this is the most widely used method in previously published studies [9,10,11,12,13,14,15,16,17,18,19], it is prone to information bias. The study’s participation criteria of births within the past six years aimed to limit this by reducing time of recall. Nonetheless, as a non-differential misclassification, it lends to observed associations towards the null.

Outcome data were obtained from birth cards and were therefore less susceptible to information bias. Measurements had been previously recorded by healthcare professionals who were unaware of this study. In Sri Lanka, access to public health care is widespread and 99.5% of births are facility based, prompting the attainment of reliable and inclusive data [26]. Where healthcare does not reach rural communities, selection bias would underestimate the association between HAP and LBW. This presented a limitation in previous studies conducted in other LMICs such as India and Pakistan [10,39]. A high number of home births in previous studies also resulted in a time lag between delivery and birth weight measurements, reducing data accuracy [10,13].

Another strength of this study was the consideration of SGA as an outcome of adverse fetal growth. Most prior studies limited outcome assessment to LBW. The prevalence of SGA was determined at 43%, over three times that of LBW, and is consistent with figures worldwide [40]. This difference suggests that LBW alone cannot fully account for all cases of adverse fetal growth, and that disregarding SGA would underestimate the potential burden of pregnancies impacted by HAP. Although this study has many strengths, future research may focus on objective measurements of indoor air pollutants, and other potential risk factors associated with indoor air pollution such as asthma and ear infections in children.

### 4.3. Policy Implications

It is no surprise that the burden of adverse fetal growth falls heavily upon the developing world. In fact, over 95% of the worlds’ LBW children are born in LMICs, and South Asia accounts for approximately half of them [41]. HAP-induced morbidities reduce productivity and perpetuate poverty further. Women often assume household cooking activities; therefore, the disproportionate burden of HAP is also an issue of gender inequality [42]. These equity implications provide strong incentives to address the impact of biomass fuels for cooking on antenatal health worldwide.

In Sri Lanka, improvements in maternal and child health have plateaued [26]. In fact, the prevalence of LBW is 16.6% and has remained stagnant for the past 20 years [5,22]. It is clear that Sri Lanka must divert attention to other factors influencing antenatal health, such as HAP. Unfortunately, HAP is not prominent in Sri Lanka’s current public health policies [43]. As evidence for the impact of HAP on birth outcomes continues to mount, there is significant opportunity to address the modifiable risk factor.

Ultimately, to mitigate the impact of exposure to biomass pollutants on fetal growth, a transition to clean energy is recommended. Unfortunately, many LMICs, including Sri Lanka, are far behind in achieving these targets due to economic, infrastructural, logistic, and cultural constraints. Therefore, as economic barriers mean a switch to clean energy is not always possible, results from this study show that improved mud stoves and increased kitchen ventilation present an alternate avenue to reduce HAP exposure until widespread access to clean energy is possible. Exposure may be further mitigated through community-based programs that aim to increase awareness of HAP and stimulate behavioural changes.

## 5. Conclusions

Results from this study show that exposure to HAP from the use of biomass fuel during pregnancy is associated with an increased risk of LBW and SGA compared to the use of clean energies. Traditional biomass stoves and kitchens without a chimney increased this risk further.

To improve antenatal health prospects, findings should stimulate policy and behavioural change for clean energy adoption amongst communities and government organizations. With a high prevalence of biomass use in Sri Lanka and across the developing world, results from this study have implications on both domestic and global scales.

## Figures and Tables

**Table 1:** Household demographics according to the primary source of cooking fuel used during pregnancy by 385 mothers in Central Sri Lanka.

	Clean Energy (Unexposed) *n* = 199 (51.69%)	Biomass (Exposed) *n* = 186 (48.31%)	Total *n* = 385, (%)
**Area, *n* (%)**			
Rural	172 (55.31)	139 (44.69)	311 (80.78)
Urban	27 (36.49)	47 (63.51)	74 (19.22)
**Education level of mother, *n* (%)**			
Primary (Grades 1–6)	3 (50.00)	3 (50.00)	6 (1.56)
Secondary (Grades 7–12)	80 (41.45)	113 (58.55)	193 (50.26)
Tertiary (University level)	116 (62.70)	69 (37.30)	185 (48.18)
**Occupation of mother, *n* (%)**			
Housewife	145 (50.35)	143 (49.65)	288 (75.99)
Day labour	9 (37.50)	15 (62.50)	24 (6.33)
Pink collar *	15 (53.57)	13 (46.43)	28 (7.39)
White collar **	18 (78.26)	5 (21.74)	23 (6.07)
Other ***	9 (56.25)	7 (43.75)	16 (4.22)
Monthly income (LKR ****)**, *n* (%)**			
≤25,000 (≤USD 132)	36 (39.13)	56 (60.87)	92 (24.53)
25,001–50,000 (USD 132–263)	107 (53.77)	92 (46.23)	199 (53.07)
50,001–75,000 (USD 263–395)	24 (50.00)	24 (50.00)	48 (12.80)
≥ 75,001 (≥USD 395)	27 (75.00)	9 (25.00)	36 (9.60)
**Average number of household members, ** $\overset{-}{x}$ **(SD)**	4.87 (1.27)	5.35 (1.29)	5.11 (1.30)

* pink collar jobs include nurse, teacher, receptionist, florist, beautician, tailor; ** white collar jobs include accountant, doctor, pharmacist, officer, advisor, clerk; *** other jobs include business/shop owner, police force; **** Sri Lankan Rupee.

**Table 2:** Primary and secondary cooking fuels and stove types used during pregnancy by 385 mothers in Central Sri Lanka.

	Primary Use, (%) *n* = 384 (100)	Secondary Use, (%) *n* = 331 (86.18) **
**Stove type**		
Gas	198 (51.56)	116 (30.21)
Electric	1 (0.26)	67 (17.45)
Traditional Biomass	83 (21.62)	57 (14.84)
Improved Biomass	102 (26.56)	91 (23.70)
Fuel type *		
Biomass		
Firewood	185 (48.18)	147 (38.28)
Coconut shells/husks	84 (21.88)	55 (14.32)
Sawdust	1 (0.26)	-
Rice husks	-	1 (0.26)
Total biomass	185 (48.18)	148 (38.54)
Clean energy		
LPG	197 (51.30)	117 (30.47)
Biogas	1 (0.26)	-
Electricity	1 (0.26)	77 (20.05)
Total clean energy	199 (51.82)	183 (47.66)

* Percentages for the type of biomass fuel do not add up because some households used more than one source of fuel for the same stove type. ** Percentages in this column are specific the total number of households.

**Table 3:** The impact of household air pollution (HAP) as determined by primary fuel type, stove type and cooking fuel, and ventilation during pregnancy on low birth weight (LBW) for 445 births in rural Sri Lanka, 2019.

	Unadjusted		Adjusted *	
Exposure to HAP	OR	95% CI	aOR	95% CI
Primary fuel *				
Biomass	1.61	0.91–2.85	2.74	1.08–6.96
Clean energy (ref)	1.00		1.00	
Primary stove type *				
Traditional Biomass	1.87	0.95–3.67	3.23	1.17–8.89
Improved Biomass	1.42	0.72–2.78	2.11	0.80–5.53
Clean stove (ref)	1.00		1.00	
Primary fuel type and kitchen ventilation**				
Very High	3.98	1.50–10.58	4.63	1.54–13.93
High	1.42	0.54–3.71	1.21	0.41–3.53
Moderate	1.41	0.54–3.73	1.19	0.41–3.43
Low (ref)	1.00		1.00	

* adjusted for income, education, area, incense, vaporiser, second-hand tobacco smoke, and chimney; ** adjusted for income, education, area, incense, vaporiser, and second-hand tobacco smoke.

**Table 4:** The impact of HAP as determined by primary fuel type, stove type and cooking fuel, and ventilation during pregnancy on the small for gestational age (SGA) parameter for 445 births in rural Sri Lanka, 2019.

	Unadjusted		Adjusted *	
Exposure to HAP	OR	95% CI	aOR	95% CI
Primary fuel *				
Biomass	1.75	1.17–2.62	1.87	1.03–3.41
Clean energy (ref)	1.00		1.00	
Primary stove type *				
Traditional Biomass	2.39	1.41–3.80	2.64	1.27–4.91
Improved Biomass	1.39	0.86–2.25	1.26	0.78–3.06
Clean energy stove—gas or electric (ref)	1.00		1.00	
Primary fuel type and kitchen ventilation **				
Very High	2.15	1.05–4.43	1.76	0.75–4.13
High	1.68	0.93–3.07	1.60	0.80–3.21
Moderate	1.02	0.56–1.88	1.01	0.50–2.04
Low (ref)	1.00		1.00	

* adjusted for income, education, area, incense, vaporiser, second-hand tobacco smoke, and chimney; ** adjusted for income, education, area, incense, vaporiser, and second-hand tobacco smoke.

## Data Availability

The data presented in this study are available in supplementary material.

## Supplementary Materials

The following are available online at https://www.mdpi.com/1660-4601/18/4/1878/s1, Figure S1: Directed Acyclic Graph of confounders for the relationship between Hap and impaired fetal growth, Table S1: Household characteristics and maternal cooking habits during pregnancy for 445 live births in Central Sri Lanka in the past 6 years, Table S2: Maternal and child health indices according to the primary fuel used during pregnancy for 445 live births in Central Sri Lanka in the past 6 years, Table S3: Secondary fuels used based on primary fuel type.
