# Supplementary material for: Household Air Pollution from Biomass Fuel for Cooking and Adverse Fetal Growth Outcomes in Rural Sri Lanka
Source: Int J Environ Res Public Health. 2021 Feb 15;18(4):1878. doi: 10.3390/ijerph18041878 (PMC7918999; doi:10.3390/ijerph18041878)
Supplement: Supplementary file 1 [file ijerph-18-01878-s001.pdf]

International Journal of
Environmental Research and Public Health

MDPI

Supplementary Materials:

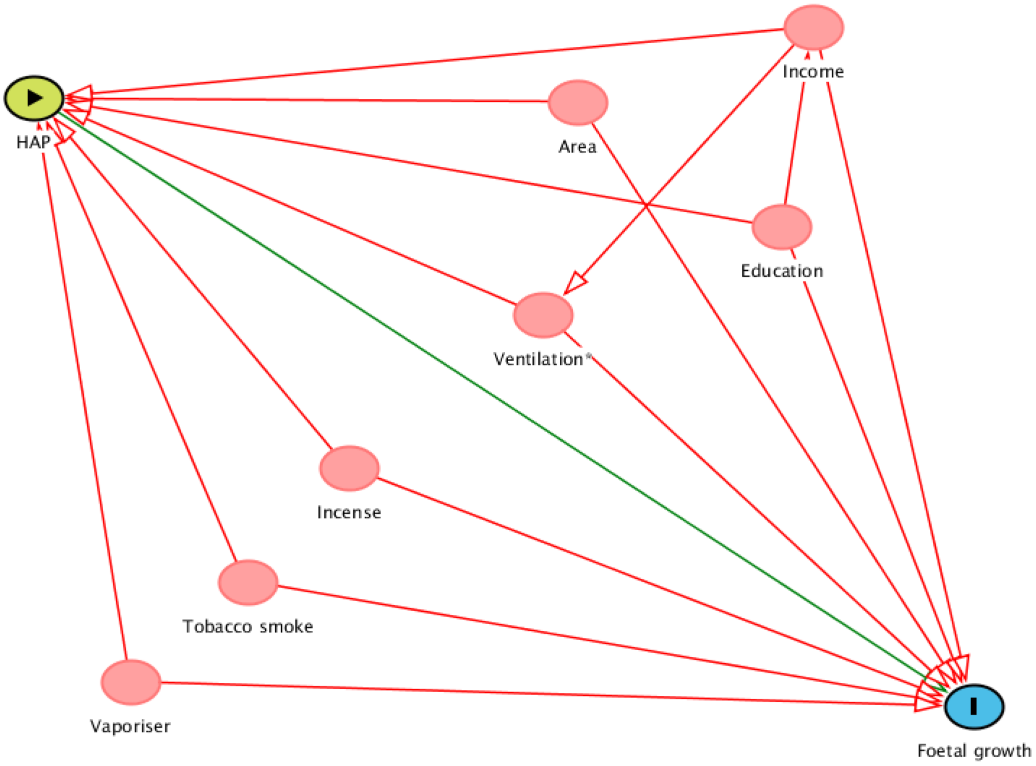

Figure S1. Directed Acyclic Graph of confounders for the relationship between Hap and impaired fetal growth.

Table S1. Household characteristics and maternal cooking habits during pregnancy for 445 live births in Central Sri Lanka in the past 6 years.

	N	%
**Hours devoted to cooking daily**		
< 2 hours	46	10.34
2-3 hours	268	60.22
>3 hours	131	29.44
**Trimesters during which mother engaged in cooking**		
One trimester	22	5.95
Two trimesters	57	15.41
All	291	78.65
**Kitchen ventilation for biomass households**		
No chimney	64	30.05
Chimney	149	69.95
No windows	49	22.90
Windows	165	77.10
**Source of lighting**		
Electricity	443	99.77
Kerosene	1	0.23
**Other sources of household air pollution**		
Incense	369	83.11
Vaporizer	17	3.90
First-hand tobacco smoke	0	0.00
Second-hand tobacco smoke	46	10.50

Table S2. Maternal and child health indices according to the primary fuel used during pregnancy for 445 live births in Central Sri Lanka in the past 6 years.

	Clean energy (Unexposed) N = 230 (51.69%)	Biomass (Exposed) N = 215 (48.31%)	Total N=445 n (%)
**Mother's age in years at time of childbirth, n (%)**			
≤21	16 (39.02)	25 (60.98)	41 (9.21)
22-34	179 (53.27)	157 (46.73)	336 (75.51)
≥35	35 (51.47)	33 (48.53)	68 (15.28)
**Mode of delivery, n (%)**			
Normal	115 (46.75)	131 (53.25)	246 (55.28)
Normal using forceps	5 (62.50)	3 (37.50)	8 (1.80)
Caesarean section	109 (57.37)	81 (42.63)	190 (42.70)
**Gravidity, n (%)**			
Primigravida	65 (51.18)	62 (48.82)	127 (28.54)
Multigravida	160 (52.12)	147 (47.88)	307 (68.99)
**Maternal health complications, n (%)**			
Anemia	22 (45.83)	26 (54.17)	48 (10.79)
Hypertension	12 (50.00)	12 (50.00)	24 (5.40)
Diabetes	18 (58.06)	13 (41.94)	31 (6.97)
**Maternal weight, n (%)**			
Underweight	12 (40.00)	18 (60.00)	30 (6.75)
Normal weight	201 (52.34)	183 (47.66)	384 (69.66)
Overweight	17 (54.84)	14 (45.16)	31 (6.97)
**Gender of child, n (%)**			
Male	133 (55.19)	108 (44.81)	241 (54.26)
Female	97 (47.55)	107 (52.45)	204 (45.84)
**Birth outcomes**			
LBW	24 (41.38)	34 (58.62)	58 (13.03)
SGA	79 (44.13)	100 (55.87)	179 (43.13)
Pre-term birth	21 (63.64)	12 (36.36)	33 (7.42)
**Child measurements at birth, $\bar{x}$ (SD)**			
Birthweight (kg)	2.69 (0.47)	2.89 (0.42)	2.92
Gestation period (weeks)	38.62 (2.32)	39.02 (1.82)	38.81

Table S3. Secondary fuels used based on primary fuel type.

	Secondary stove				
Primary stove (N)	Gas	Electric	Traditional Biomass	Improved Biomass	Kerosene
Gas (198)	0	40	49	85	0
Electric (1)	0	0	1	0	0
Traditional Biomass (83)	57	9	0	0	0
Improved Biomass (102)	65	18	7	0	1

Annexure I

The association between household air pollution on respiratory disease and pregnancy outcomes in Sri Lankan communities

Today's date: ______________________

Interviewer: ______________________

Survey ID: ______________________

Home address: ______________________

MoH and Village: ______________________

Contact number: ______________________

GPS location: ______________________

The purpose of this questionnaire is to investigate household fuel use for cooking and its health and environmental effects. It is for research purpose only. Please answer the questions to the best of your knowledge. Answers will be kept completely confidential and only be presented in summary formats.

Consented to the study? ☐ Yes ☐ No

SECTION A: Household Socio-Economic Characteristics	
A1. How many people normally live in this home/ house?	______________
A2. What is the monthly income of the family?	______________ LKR/ month

SECTION C: Stove Characteristics

C1. What is the primary stove type?	☐ Gas ☐ Electric ☐ Kerosene<br>☐ Mud Traditional ☐ Mud Improved<br>☐ Other (specify) _ _ _ _ _ _ _ _ _ _ _ _ _
C2. Which fuels are used?<br>Please tick all that apply	☐ Firewood ☐ Sawdust ☐ Tree residue<br>☐ Straw ☐ Rice husk ☐ Coconut husks/leaves<br>☐ Animal residue ☐ Charcoal ☐ Kerosene<br>☐ LPG ☐ Bio gas ☐ Electricity<br>☐ Coconut shells<br>☐ Other (specify) _ _ _ _ _ _ _ _ _ _ _ _ _
C3. Does the stove have a chimney?	☐ Yes ☐ No
C4. Do you have a secondary stove?<br>If no skip to question C8	☐ Yes ☐ No
C5. What type of stove is it?	☐ Gas ☐ Electric ☐ Kerosene<br>☐ Mud Traditional ☐ Mud Improved<br>☐ Other (specify) _ _ _ _ _ _ _ _ _ _ _ _ _
C6. Which fuels are used?<br>Please tick all that apply	☐ Firewood ☐ Sawdust ☐ Tree residue<br>☐ Straw ☐ Rice husk ☐ Coconut husks/leaves<br>☐ Animal residue ☐ Charcoal ☐ Kerosene<br>☐ LPG ☐ Bio gas ☐ Electricity<br>☐ Coconut shell<br>☐ Other (specify) _ _ _ _ _ _ _ _ _ _ _ _ _

C7. Does the stove have a chimney?	☐ Yes ☐ No
C8. Do you keep windows/doors of the kitchen open when cooking?	☐ Yes ☐ No

SECTION D: Other Sources of Pollutants

D1. Do you use mosquito coils inside the house?<br>If no skip to question D3	☐ Yes ☐ No
D2. How frequently do you use mosquito coils?	☐ Daily<br>☐ 1-2 times a week<br>☐ 3 or more times a week ☐ Rarely
D3. Do you burn incense inside your house?<br>If no skip to question D5	☐ Yes ☐ No
D4. How frequently do you burn incense inside your house?	☐ Daily<br>☐ 1-2 times a week<br>☐ 3 or more times a week ☐ Rarely
D5. Do you mosquito repellent vaporizers inside your house?<br>If no skip to question D7	☐ Yes ☐ No
D6. How frequently do you use vaporizers inside your house?	☐ Daily<br>☐ 1-2 times a week<br>☐ 3 or more times a week ☐ Rarely
D7. What is the main source of lighting used for your household?	☐ Electricity ☐ Kerosene lamp<br>☐ Other (specify) _ _ _ _ _ _ _ _ _ _ _ _

SECTION E: Background Information

For all children ≤ 5 years and younger and adults ≥18 years living in the house

Person ID				
E1. What is your name?	First:<br>Last:	First:<br>Last:	First:<br>Last:	First:<br>Last:
E2. What is your gender?	☐ Male<br>☐ Female<br>☐ Other/not specified	☐ Male<br>☐ Female<br>☐ Other/not specified	☐ Male<br>☐ Female<br>☐ Other/not specified	☐ Male<br>☐ Female<br>☐ Other/not specified
E3. What is your date of birth and age in years?	DOB: dd/mm/yyyy<br>Age: _ _ _ _ _ _ years	DOB: dd/mm/yyyy<br>Age: _ _ _ _ _ _ years	DOB: dd/mm/yyyy<br>Age: _ _ _ _ _ _ years	DOB: dd/mm/yyyy<br>Age: _ _ _ _ _ _ years
E4. Adults: What is your highest education level?	☐ No schooling<br>☐ ≤ Grade 5 schooling<br>☐ Grade 6-10 schooling<br>☐ Grade 11-12 schooling<br>☐ > Grade 12 schooling<br>☐ Certificate/Diploma<br>☐ Undergraduate<br>☐ Graduate	☐ No schooling<br>☐ ≤ Grade 5 schooling<br>☐ Grade 6-10 schooling<br>☐ Grade 11-12 schooling<br>☐ > Grade 12 schooling<br>☐ Certificate/Diploma<br>☐ Undergraduate<br>☐ Graduate	☐ No schooling<br>☐ ≤ Grade 5 schooling<br>☐ Grade 6-10 schooling<br>☐ Grade 11-12 schooling<br>☐ > Grade 12 schooling<br>☐ Certificate/Diploma<br>☐ Undergraduate<br>☐ Graduate	☐ No schooling<br>☐ ≤ Grade 5 schooling<br>☐ Grade 6-10 schooling<br>☐ Grade 11-12 schooling<br>☐ > Grade 12 schooling<br>☐ Certificate/Diploma<br>☐ Undergraduate<br>☐ Graduate
E5. How many years have you resided in the house?	_ _ _ _ _ _ _ _ _ years	_ _ _ _ _ _ _ _ _ years	_ _ _ _ _ _ _ _ _ years	_ _ _ _ _ _ _ _ _ years
E6. Adults: What is your main occupation? Please tick.	☐ House wife<br>☐ Unemployed<br>☐ Farmer<br>☐ Day labor	☐ House wife<br>☐ Unemployed<br>☐ Farmer<br>☐ Day labor	☐ House wife<br>☐ Unemployed<br>☐ Farmer<br>☐ Day labor	☐ House wife<br>☐ Unemployed<br>☐ Farmer<br>☐ Day labor

	☐ Self-employed<br>☐ Student<br>☐ Disabled<br>☐ Others (please specify) ____________	☐ Self-employed<br>☐ Student<br>☐ Disabled<br>☐ Others (please specify) ____________	☐ Self-employed<br>☐ Student<br>☐ Disabled<br>☐ Others (please specify) ____________	☐ Self-employed<br>☐ Student<br>☐ Disabled<br>☐ Others (please specify) ____________
E7. If applicable what is your secondary occupation? (use code mentioned for primary occupation)	☐ House wife<br>☐ Unemployed<br>☐ Farmer<br>☐ Day labor<br>☐ Self-employed<br>☐ Student<br>☐ Disabled<br>☐ Others (please specify) ____________	☐ House wife<br>☐ Unemployed<br>☐ Farmer<br>☐ Day labor<br>☐ Self-employed<br>☐ Student<br>☐ Disabled<br>☐ Others (please specify) ____________	☐ House wife<br>☐ Unemployed<br>☐ Farmer<br>☐ Day labor<br>☐ Self-employed<br>☐ Student<br>☐ Disabled<br>☐ Others (please specify) ____________	☐ House wife<br>☐ Unemployed<br>☐ Farmer<br>☐ Day labor<br>☐ Self-employed<br>☐ Student<br>☐ Disabled<br>☐ Others (please specify) ____________
E8. Each day how many hours on average do you spend in kitchen while food is being cooked?	_________ hours	_________ hours	_________ hours	_________ hours
E9. Are you a smoker?	☐ Yes<br>☐ No<br>☐ Ex-smoker	☐ Yes<br>☐ No<br>☐ Ex-smoker	☐ Yes<br>☐ No<br>☐ Ex-smoker	☐ Yes<br>☐ No<br>☐ Ex-smoker
E10. If yes, or ex-smoker how do/did you smoke?	☐ Loose tobacco<br>☐ Cigarette without filter<br>☐ Cigarette with filter	☐ Loose tobacco<br>☐ Cigarette without filter<br>☐ Cigarette with filter	☐ Loose tobacco<br>☐ Cigarette without filter<br>☐ Cigarette with filter	☐ Loose tobacco<br>☐ Cigarette without filter<br>☐ Cigarette with filter
E11. How many cigarettes per day do you/ did you smoke on average?				

	_ _ _ _ _ _ _ _ _ cigarettes	_ _ _ _ _ _ _ _ _ cigarettes	_ _ _ _ _ _ _ _ _ cigarettes	_ _ _ _ _ _ _ _ _ cigarettes

Continue…For all children ≤ 5 years and younger and adults ≥ 18 years living in the house

Person ID				
E1. What is your name?	First:<br>Last:	First:<br>Last:	First:<br>Last:	First:<br>Last:
E2. What is your gender?	☐ Male<br>☐ Female<br>☐ Other/not specified	☐ Male<br>☐ Female<br>☐ Other/not specified	☐ Male<br>☐ Female<br>☐ Other/not specified	☐ Male<br>☐ Female<br>☐ Other/not specified
E3. What is your date of birth and age in years?	DOB: dd/mm/yyyy<br>Age: _ _ _ _ _ _ years	DOB: dd/mm/yyyy<br>Age: _ _ _ _ _ _ years	DOB: dd/mm/yyyy<br>Age: _ _ _ _ _ _ years	DOB: dd/mm/yyyy<br>Age: _ _ _ _ _ _ years
E4. Adults: What is your highest education level?	☐ No schooling<br>☐ ≤ Grade 5 schooling<br>☐ Grade 6-10 schooling<br>☐ Grade 11-12 schooling<br>☐ > Grade 12 schooling<br>☐ Certificate/Diploma<br>☐ Undergraduate<br>☐ Graduate	☐ No schooling<br>☐ ≤ Grade 5 schooling<br>☐ Grade 6-10 schooling<br>☐ Grade 11-12 schooling<br>☐ > Grade 12 schooling<br>☐ Certificate/Diploma<br>☐ Undergraduate<br>☐ Graduate	☐ No schooling<br>☐ ≤ Grade 5 schooling<br>☐ Grade 6-10 schooling<br>☐ Grade 11-12 schooling<br>☐ > Grade 12 schooling<br>☐ Certificate/Diploma<br>☐ Undergraduate<br>☐ Graduate	☐ No schooling<br>☐ ≤ Grade 5 schooling<br>☐ Grade 6-10 schooling<br>☐ Grade 11-12 schooling<br>☐ > Grade 12 schooling<br>☐ Certificate/Diploma<br>☐ Undergraduate<br>☐ Graduate
E5. How many years have you resided in the house?	_ _ _ _ _ _ _ _ _ years	_ _ _ _ _ _ _ _ _ years	_ _ _ _ _ _ _ _ _ years	_ _ _ _ _ _ _ _ _ years

E6. Adults: What is your main occupation? Please tick.	☐ House wife<br>☐ Unemployed<br>☐ Farmer<br>☐ Day labor<br>☐ Self-employed<br>☐ Student<br>☐ Disabled<br>☐ Others (please specify)<br>_ _ _ _ _ _ _ _ _ _ _<br>_	☐ House wife<br>☐ Unemployed<br>☐ Farmer<br>☐ Day labor<br>☐ Self-employed<br>☐ Student<br>☐ Disabled<br>☐ Others (please specify)<br>_ _ _ _ _ _ _ _ _ _ _<br>_	☐ House wife<br>☐ Unemployed<br>☐ Farmer<br>☐ Day labor<br>☐ Self-employed<br>☐ Student<br>☐ Disabled<br>☐ Others (please specify)<br>_ _ _ _ _ _ _ _ _ _ _<br>_	☐ House wife<br>☐ Unemployed<br>☐ Farmer<br>☐ Day labor<br>☐ Self-employed<br>☐ Student<br>☐ Disabled<br>☐ Others (please specify)<br>_ _ _ _ _ _ _ _ _ _ _<br>_
E7. If applicable what is your secondary occupation? (use code mentioned for primary occupation)	☐ House wife<br>☐ Unemployed<br>☐ Farmer<br>☐ Day labor<br>☐ Self-employed<br>☐ Student<br>☐ Disabled<br>☐ Others (please specify)<br>_ _ _ _ _ _ _ _ _ _ _<br>_	☐ House wife<br>☐ Unemployed<br>☐ Farmer<br>☐ Day labor<br>☐ Self-employed<br>☐ Student<br>☐ Disabled<br>☐ Others (please specify)<br>_ _ _ _ _ _ _ _ _ _<br>_ _	☐ House wife<br>☐ Unemployed<br>☐ Farmer<br>☐ Day labor<br>☐ Self-employed<br>☐ Student<br>☐ Disabled<br>☐ Others (please specify)<br>_ _ _ _ _ _ _ _ _ _ _<br>_	☐ House wife<br>☐ Unemployed<br>☐ Farmer<br>☐ Day labor<br>☐ Self-employed<br>☐ Student<br>☐ Disabled<br>☐ Others (please specify)<br>_ _ _ _ _ _ _ _ _ _ _<br>_
E8. Each day how many hours on average do you spend in kitchen while food is being cooked?	_ _ _ _ _ _ _ _ hours	_ _ _ _ _ _ _ _ hours	_ _ _ _ _ _ _ _ hours	_ _ _ _ _ _ _ _ hours
E9. Are you a smoker?	☐ Yes<br>☐ No<br>☐ Ex-smoker	☐ Yes<br>☐ No<br>☐ Ex-smoker	☐ Yes<br>☐ No<br>☐ Ex-smoker	☐ Yes<br>☐ No<br>☐ Ex-smoker
E10. If yes, or ex-smoker how do/did	☐ Loose tobacco	☐ Loose tobacco	☐ Loose tobacco	☐ Loose tobacco

you smoke?	☐ Cigarette without filter<br>☐ Cigarette with filter	☐ Cigarette without filter<br>☐ Cigarette with filter	☐ Cigarette without filter<br>☐ Cigarette with filter	☐ Cigarette without filter<br>☐ Cigarette with filter
E11. How many cigarettes per day do you/ did you smoke on average?	_ _ _ _ _ _ _ _ _ cigarettes	_ _ _ _ _ _ _ _ _ cigarettes	_ _ _ _ _ _ _ _ _ cigarettes	_ _ _ _ _ _ _ _ _ cigarettes

SECTION F: Characteristics of Primary Cook

Please provide information for each person who cooks in the household. By cooking we mean cooking while the stove is in use.

ID of cook				
F1. At which age did you start to cook?	_ _ _ _ _ _ _ _ _ years	_ _ _ _ _ _ _ _ _ years	_ _ _ _ _ _ _ _ _ years	_ _ _ _ _ _ _ _ _ years
F2. How many days do you cook in a week?	_ _ _ _ _ _ _ _ _ days	_ _ _ _ _ _ _ _ _ days	_ _ _ _ _ _ _ _ _ days	_ _ _ _ _ _ _ _ _ days
F3. On a typical day, how many hours do you spend cooking?	_ _ _ _ _ _ _ _ _ hours	_ _ _ _ _ _ _ _ _ hours	_ _ _ _ _ _ _ _ _ hours	_ _ _ _ _ _ _ _ _ hours
F4. Do you still engage in cooking?	☐ Yes<br>☐ No	☐ Yes<br>☐ No	☐ Yes<br>☐ No	☐ Yes<br>☐ No

SECTION G: Pregnancy Outcomes

(Questions from this section are specific to the mother)

I1. How many children have you had?	_ _ _ _ _ _ children
I2. Have you ever had a stillbirth (infant died in womb after 28 weeks of gestation)?	☐ Yes ☐ No
I3. Have you ever had an abortion (miscarriage before 3 months)?	☐ Yes ☐ No
I4. Have you ever lost a child within one week of their birth (perinatal mortality)?	☐ Yes ☐ No

(The following questions are to be answered by the mother relevant to each child under the age of 5 years)

Child ID				
I5. When you were pregnant, were you involved in cooking? If no skip to I8	☐ Yes<br>☐ No	☐ Yes<br>☐ No	☐ Yes<br>☐ No	☐ Yes<br>☐ No
I6. If yes, please state during which trimesters.	☐ Trimester 1<br>☐ Trimester 2<br>☐ Trimester 3<br>☐ ALL	☐ Trimester 1<br>☐ Trimester 2<br>☐ Trimester 3<br>☐ ALL	☐ Trimester 1<br>☐ Trimester 2<br>☐ Trimester 3<br>☐ ALL	☐ Trimester 1<br>☐ Trimester 2<br>☐ Trimester 3<br>☐ ALL
I7. If yes how many hours did you spending cooking on average each day?	☐ <1 hour per day<br>☐ 1-2 hours per day<br>☐ 2-5 hours per day<br>☐ >5 hours per day	☐ <1 hour per day<br>☐ 1-2 hours per day<br>☐ 2-5 hours per day<br>☐ >5 hours per day	☐ <1 hour per day<br>☐ 1-2 hours per day<br>☐ 2-5 hours per day<br>☐ >5 hours per day	☐ <1 hour per day<br>☐ 1-2 hours per day<br>☐ 2-5 hours per day<br>☐ >5 hours per day
I8. When you were pregnant did you use mosquito coils inside the house? If no skip to I10	☐ Yes<br>☐ No	☐ Yes<br>☐ No	☐ Yes<br>☐ No	☐ Yes<br>☐ No

I9. If yes how often did you use mosquito coils?	☐ Daily<br>☐ 1-2 times a week<br>☐ 3 or more times a week<br>☐ Rarely	☐ Daily<br>☐ 1-2 times a week<br>☐ 3 or more times a week<br>☐ Rarely	☐ Daily<br>☐ 1-2 times a week<br>☐ 3 or more times a week<br>☐ Rarely	☐ Daily<br>☐ 1-2 times a week<br>☐ 3 or more times a week<br>☐ Rarely
I10. When you were pregnant did you use incense inside the house? If no skip to I12	☐ Yes<br>☐ No	☐ Yes<br>☐ No	☐ Yes<br>☐ No	☐ Yes<br>☐ No
I11. If yes how often did you use incense?	☐ Daily<br>☐ 1-2 times a week<br>☐ 3 or more times a week<br>☐ Rarely	☐ Daily<br>☐ 1-2 times a week<br>☐ 3 or more times a week<br>☐ Rarely	☐ Daily<br>☐ 1-2 times a week<br>☐ 3 or more times a week<br>☐ Rarely	☐ Daily<br>☐ 1-2 times a week<br>☐ 3 or more times a week<br>☐ Rarely
I12. When you were pregnant did you use vaporizers inside the house? If no skip to I14	☐ Yes<br>☐ No	☐ Yes<br>☐ No	☐ Yes<br>☐ No	☐ Yes<br>☐ No
I13. If yes how often did you use vaporizers?	☐ Daily<br>☐ 1-2 times a week<br>☐ 3 or more times a week<br>☐ Rarely	☐ Daily<br>☐ 1-2 times a week<br>☐ 3 or more times a week<br>☐ Rarely	☐ Daily<br>☐ 1-2 times a week<br>☐ 3 or more times a week<br>☐ Rarely	☐ Daily<br>☐ 1-2 times a week<br>☐ 3 or more times a week<br>☐ Rarely
I14. Did you drink alcohol during pregnancy? If no skip to I16	☐ Yes<br>☐ No	☐ Yes<br>☐ No	☐ Yes<br>☐ No	☐ Yes<br>☐ No

I15. If yes how many times did you drink on an average week?	☐ Daily<br>☐ 1-2 times a week<br>☐ 3 or more times a week<br>☐ Rarely	☐ Daily<br>☐ 1-2 times a week<br>☐ 3 or more times a week<br>☐ Rarely	☐ Daily<br>☐ 1-2 times a week<br>☐ 3 or more times a week<br>☐ Rarely	☐ Daily<br>☐ 1-2 times a week<br>☐ 3 or more times a week<br>☐ Rarely
I16. Did you smoke tobacco during pregnancy? If no skip to I18	☐ Yes<br>☐ No	☐ Yes<br>☐ No	☐ Yes<br>☐ No	☐ Yes<br>☐ No
I17. If yes how many times did you smoke on average each day?	☐ Daily<br>☐ 1-2 times a week<br>☐ 3 or more times a week<br>☐ Rarely	☐ Daily<br>☐ 1-2 times a week<br>☐ 3 or more times a week<br>☐ Rarely	☐ Daily<br>☐ 1-2 times a week<br>☐ 3 or more times a week<br>☐ Rarely	☐ Daily<br>☐ 1-2 times a week<br>☐ 3 or more times a week<br>☐ Rarely
I18. Did anyone else in your household smoke tobacco whilst you were pregnant? If no skip to I20	☐ Yes<br>☐ No	☐ Yes<br>☐ No	☐ Yes<br>☐ No	☐ Yes<br>☐ No
I19. If yes how many times were you exposed to the smoke on average each week?	☐ Daily<br>☐ 1-2 times a week<br>☐ 3 or more times a week<br>☐ Rarely	☐ Daily<br>☐ 1-2 times a week<br>☐ 3 or more times a week<br>☐ Rarely	☐ Daily<br>☐ 1-2 times a week<br>☐ 3 or more times a week<br>☐ Rarely	☐ Daily<br>☐ 1-2 times a week<br>☐ 3 or more times a week<br>☐ Rarely

I20. Did you have any of the following health complications during pregnancy? Please tick all that apply, and state any not listed.	☐ Anemia<br>☐ Malnutrition<br>☐ Obesity<br>☐ Diabetes<br>☐ Hypertension<br>☐ Dengue fever<br>☐ HIV/AIDs<br>☐ Kidney disease<br>☐ Thyroid disease<br>☐ Other (specify)<br>_ _ _ _ _ _ _ _ _ _<br>_ _ _ _ _ _ _ _ _ _	☐ Anemia<br>☐ Malnutrition<br>☐ Obesity<br>☐ Diabetes<br>☐ Hypertension<br>☐ Dengue fever<br>☐ HIV/AIDs<br>☐ Kidney disease<br>☐ Thyroid disease<br>☐ Other (specify)<br>_ _ _ _ _ _ _ _ _ _<br>_ _ _ _ _ _ _ _ _ _	☐ Anemia<br>☐ Malnutrition<br>☐ Obesity<br>☐ Diabetes<br>☐ Hypertension<br>☐ Dengue fever<br>☐ HIV/AIDs<br>☐ Kidney disease<br>☐ Thyroid disease<br>☐ Other (specify)<br>_ _ _ _ _ _ _ _ _ _<br>_ _ _ _ _ _ _ _ _ _	☐ Anemia<br>☐ Malnutrition<br>☐ Obesity<br>☐ Diabetes<br>☐ Hypertension<br>☐ Dengue fever<br>☐ HIV/AIDs<br>☐ Kidney disease<br>☐ Thyroid disease<br>☐ Other (specify)<br>_ _ _ _ _ _ _ _ _ _<br>_ _ _ _ _ _ _ _ _ _

(The following questions are to be answered using birth cards for each child under the age of 5 years)

Child ID				
I21. Was child part of a multiple pregnancy i.e. a twin?	☐ Yes<br>☐ No	☐ Yes<br>☐ No	☐ Yes<br>☐ No	☐ Yes<br>☐ No
I22. What is the gender of the child?	☐ Male<br>☐ Female	☐ Male<br>☐ Female	☐ Male<br>☐ Female	☐ Male<br>☐ Female
I23. What was the mother's age at time of child birth?	_ _ _ _ _ _ _ _ _ years	_ _ _ _ _ _ _ _ _ years	_ _ _ _ _ _ _ _ _ years	_ _ _ _ _ _ _ _ _ years
I24. What was the mode of child delivery?	☐ Normal<br>☐ Normal using forceps<br>☐ Using vacuum<br>☐ Caesarean section	☐ Normal<br>☐ Normal using forceps<br>☐ Using vacuum<br>☐ Caesarean section	☐ Normal<br>☐ Normal using forceps<br>☐ Using vacuum<br>☐ Caesarean section	☐ Normal<br>☐ Normal using forceps<br>☐ Using vacuum<br>☐ Caesarean section
I25. What was the birth weight of the child in kg?	_ _ _ _ _ _ _ _ _ kg	_ _ _ _ _ _ _ _ _ kg	_ _ _ _ _ _ _ _ _ kg	_ _ _ _ _ _ _ _ _ kg
I26. What was the child's height at the				

time of birth in cm?	_ _ _ _ _ _ _ _ _ cm	_ _ _ _ _ _ _ _ _ cm	_ _ _ _ _ _ _ _ _ cm	_ _ _ _ _ _ _ _ _ cm
I27. What was the head-circumference of the child at the time of birth in cm?	_ _ _ _ _ _ _ _ _ cm	_ _ _ _ _ _ _ _ _ cm	_ _ _ _ _ _ _ _ _ cm	_ _ _ _ _ _ _ _ _ cm
I28. Was the child born preterm?	☐ Yes<br>☐ No	☐ Yes<br>☐ No	☐ Yes<br>☐ No	☐ Yes<br>☐ No
I29. What was the gestation period in weeks? (interviewee to ask mother as not on birth card)	_ _ _ _ _ _ _ _ _ weeks	_ _ _ _ _ _ _ _ _ weeks	_ _ _ _ _ _ _ _ _ weeks	_ _ _ _ _ _ _ _ _ weeks
I30. Did the child have neonatal complications?	☐ Yes<br>☐ No	☐ Yes<br>☐ No	☐ Yes<br>☐ No	☐ Yes<br>☐ No
I31. Did the child have congenital abnormalities?	☐ Yes<br>☐ No	☐ Yes<br>☐ No	☐ Yes<br>☐ No	☐ Yes<br>☐ No
I32. Did the child have congenital hypothyroidism?	☐ Yes<br>☐ No	☐ Yes<br>☐ No	☐ Yes<br>☐ No	☐ Yes<br>☐ No
I33. Did neonatal examination indicate any abnormalities? (If "yes" please state as on the birth card)	☐ Yes _ _ _ _ _ _ _ _ _<br>☐ No	☐ Yes _ _ _ _ _ _ _ _ _<br>☐ No	☐ Yes _ _ _ _ _ _ _ _ _<br>☐ No	☐ Yes _ _ _ _ _ _ _ _ _<br>☐ No
